# Complete Genome Sequence of Biocontroller *Bacillus velezensis* Strain JTYP2, Isolated from Leaves of *Echeveria laui*

**DOI:** 10.1128/genomeA.00505-17

**Published:** 2017-06-15

**Authors:** Beibei Wang, Hu Liu, Hailin Ma, Chengqiang Wang, Kai Liu, Yuhuan Li, Qihui Hou, Ruofei Ge, Tongrui Zhang, Fangchun Liu, Jinjin Ma, Yun Wang, Haide Wang, Baochao Xu, Gan Yao, Wenfeng Xu, Lingchao Fan, Yanqin Ding, Binghai Du

**Affiliations:** a College of Life Sciences, Shandong Key Laboratory of Agricultural Microbiology, National Engineering Laboratory for Efficient Utilization of Soil and Fertilizer Resources, Shandong Agricultural University, Tai’an, China; b Shandong Academy of Forestry, Jinan, China; c College of Resources and Environment, Shandong Agricultural University, Tai’an, China; d State Key Laboratory of Nutrition Resources Integrated Utilization, Linshu, China

## Abstract

*Bacillus velezensis* JTYP2 was isolated from the leaves of* Echeveria laui* in Qingzhou, China, and may control some of the fungal pathogens of the plant. Here, we present the complete genome sequence of *B. velezensis* JTYP2. Several gene clusters related to its biosynthesis of antimicrobial compounds were predicted.

## GENOME ANNOUNCEMENT

To decrease the pesticide residue and environmental pollution in agricultural production, more and more bacteria are being applied as biological agents to suppress plant pathogens or promote plant growth (1–3). *Bacillus velezensis* is reported to be one of the plant growth-promoting bacteria. It has been reclassiﬁed as a synonym of *B. methylotrophicus*, *B. amyloliquefaciens* subsp. *plantarum*, and *B. oryzicola* (4). Some characteristics of *B. velezensis* for plant-growth promotion have been identified. Meng et al. reported that *B. velezensis* BAC03 could promote the growth of some plants through IAA production, NH_3_ production, and ACC-deaminase activity (5). *B. velezensis* Bve2 can promote the growth of cotton and reduce the population density of *Meloidogyne incognita* (6). *B. velezensis* RC218 has biocontrol effects on *Fusarium* head blight (7).

Recently, *B. velezensis* strain JTYP2 was isolated from the leaves of *Echeveria laui* in Qingzhou, China. It exhibits strong inhibition against *Fusarium inflexum*, which can cause black rot disease of *Echeveria laui*. To more fully understand the molecular genetic characteristics of this strain, the complete genome sequence was obtained. A high-quality genomic DNA was extracted, randomly fragmented, and then sequenced using the PacBio platform. Single-molecule real-time (SMRT) DNA sequencing of 10 kb was carried out (8). The sequences were *de novo* assembled by SmrtLink (9) (v3.1.1). The genome coverage of *B. velezensis* JTYP2 reached 980×. The genome was annotated by the NCBI Prokaryotic Genome Annotation Pipeline (PGAP) (https://www.ncbi.nlm.nih.gov/genome/annotation_prok/). The repeated sequences were detected by RepeatModeler (10) (v1.0.8). In addition, the gene clusters involved in the biosynthesis of secondary metabolites were predicted by antiSMASH (11) (v3.0.5, http://antismash.secondarymetabolites.org/).

The circular chromosome of* B. velezensis* JTYP2 consists of 3,929,789 bp, with a G+C content of 46.5%. A total of 3,889 genes were annotated, among which, 3,656 coding genes were involved. The number of RNA genes in the genome was 118, including 27 rRNA genes, 86 tRNA genes, and 5 noncoding RNA (ncRNA) genes. Meanwhile, 115 pseudo genes were annotated. There were 7 short interspersed nuclear elements (SINEs), 25 long interspersed nuclear elements (LINEs), 3 long terminal repeats (LTRs), and 13 transposable elements. A total of 12 gene clusters were predicted to code antagonistic substances on plant pathogens, and half of them present high similarity with the known gene clusters. Two gene clusters (BAJT_07230-BAJT_07470 and BAJT_11035-BAJT_11300), which belong to type transAT polyketide synthase (PKS), were similar to the biosynthetic genes of macrolactin and difficidin, respectively. Two gene clusters (BAJT_08585-BAJT_08825 and BAJT_09175-BAJT_09505) were classified as nonribosomal peptide synthetase (NRPS) type transAT PKSs. The first one showed 100% similarity with the biosynthetic genes of bacillaene. The other one showed 100% similarity with the gene cluster of fengycin. An NRPS-bacteriocin type gene cluster (BAJT_14725-BAJT_15045) was related to bacillibactin biosynthesis. A gene cluster (BAJT_17730-BAJT_17945) was detected to be relevant to bacilysin production. The other 6 clusters of genes might be involved in biosynthesis of new antimicrobial compounds. The complete genome data will be helpful to understand the molecular mechanisms of biocontrol in *B. velezensis* JTYP2.

### Accession number(s).

The genome sequence of *Bacillus velezensis* JTYP2 has been deposited in GenBank under the accession number CP020375. The version described in this paper is the first version, CP020375.1.
